# Severe Maternal Morbidity and Mortality in Sickle Cell Disease in the National Inpatient Sample, 2012-2018

**DOI:** 10.1001/jamanetworkopen.2022.54552

**Published:** 2023-02-02

**Authors:** Macy L. Early, Ahizechukwu C. Eke, Alison Gemmill, Sophie Lanzkron, Lydia H. Pecker

**Affiliations:** 1 Division of Hematology, Department of Medicine, Johns Hopkins University School of Medicine, Baltimore, Maryland; 2 Division of Maternal-Fetal Medicine & Clinical Pharmacology, Department of Gynecology and Obstetrics, Johns Hopkins University School of Medicine, Baltimore, Maryland; 3 Department of Population, Family and Reproductive Health, Johns Hopkins Bloomberg School of Public Health, Baltimore, Maryland; 4 Division of Hematology, Department of Medicine, Johns Hopkins University School of Medicine, Baltimore, Maryland

## Abstract

**Importance:**

Pregnancy outcomes are historically poor among people with sickle cell disease (SCD) in the US, most of whom have Black race. Whether outcomes have improved is unknown.

**Objective:**

To tabulate adverse pregnancy outcomes among patients with SCD, comparing outcomes of deliveries among Black people with SCD with those of Black people without SCD and a control non-Black population, and to measure the association of racial disparities with adverse outcomes in SCD pregnancies.

**Design, Setting, and Participants:**

This cross-sectional study was a secondary analysis involving data from National Inpatient Sample, a nationally representative sample of 20% of acute hospital admissions in the US, between 2012 and 2018. The data set included all admissions with codes for delivery of a pregnancy among people aged 11 to 55 years. Data were analyzed from September 2021 to August 2022.

**Exposures:**

SCD, racial disparities.

**Main Outcomes and Measures:**

Severe maternal morbidity (SMM) as measured by the US Centers for Disease Control and Prevention’s index alongside other outcomes; multiple logistic regression was used to compare the odds for adverse pregnancy outcomes.

**Results:**

The sample included 5 401 899 deliveries, including 3901 deliveries among people with SCD and 742 164 deliveries among people with Black race. Compared with the non-Black control group, patients with SCD and Black patients were younger (mean [SD] age: SCD, 27.2 [5.9] years; Black, 27.1 [6.1] years vs 28.7 [5.9] years) and more likely to have public insurance (SCD, 2609 deliveries [67.3%]; Black, 496 828 deliveries [65.4%] vs 1 880 198 deliveries [40.8%]). The maternal mortality rate in deliveries among people with SCD was 26 times greater than in the non-Black control group and more than 10 times greater than among Black pregnant people without SCD (Per 10 000 deliveries: SCD 13.3; 95% CI, 5.7-31.2; Black race, 1.2; 95% CI, 1.0-1.5; non-Black control 0.5; 95% CI, 0.5-0.6). Compared with the control group, SCD deliveries had higher odds of SMM (adjusted odds ratio [aOR], 7.22; 95% CI, 6.25-8.34; *P* < .001), especially cerebrovascular events (aOR, 22.00; 95% CI, 15.25-31.72; *P* < .001) and thromboembolism (aOR, 17.34; 95% CI, 11.55-26.03; *P* < .001). Racial disparities explained a median (IQR) 28.9% (21.2%-33.1%) of the increased risk in deliveries to people with SCD and between 40% and 50% of the increased risk for acute kidney failure (excess risk [ER], 56.9%; 95% CI, 54.3%-59.3%), intrauterine fetal demise (ER, 47.8%; 95% CI, 46.6%-49.1%), and eclampsia (ER, 42.1%; 95% CI, 37.9%-46.1%).

**Conclusions and Relevance:**

In this large cross-sectional study of pregnancy outcomes in people with SCD, the risk for SMM was higher compared with deliveries among people without SCD, especially for thrombotic events, organ failure, and death. Racial disparities were associated with adverse outcomes. Our findings compel scientific, clinical, and political effort to improve outcomes for pregnant people with SCD.

## Key Points

**Question:**

What are the rates of and risks for severe maternal morbidity (SMM) among birthing people with sickle cell disease (SCD), and what proportion of the increased risk for adverse pregnancy outcomes in SCD is associated with racial disparities encountered by Black patients?

**Findings:**

In this cross-sectional study including 3901 deliveries among people with SCD, the maternal mortality rate for people with SCD was 26 times greater than in control deliveries of pregnant people with non-Black race and more than 10 times greater than deliveries among Black pregnant people. Compared with groups without SCD, deliveries among people with SCD had significantly higher odds of SMM, and racial disparities explained, on average, 28.9% of the increased risk in SCD deliveries.

**Meaning:**

These results suggest that the risk for SMM is higher in deliveries among people with SCD than those of Black or non-Black control populations with no SCD; nearly one-third of the increased risk may be attributable to racial disparities.

## Teaser

This cross-sectional study of deliveries in US hospitals examines pregnancy outcomes for people with sickle cell disease (SCD) and compares adverse outcomes with Black and Non-Black populations without SCD.

## Introduction

Pregnant people with sickle cell disease (SCD) experience pregnancy complications including eclampsia, sepsis, venous thromboembolism, and intrauterine growth restriction at much higher rates than the general population.^1,2^ Between 1999 and 2008 in the US, maternal mortality in SCD was between 7.2 and 16.0 deaths per 10 000 SCD pregnancies^1,2^ compared with 1.3 deaths per 10 000 pregnancies in the general population.^1^ Poor pregnancy outcomes may be attributable to disease pathophysiology^3^ and structural deficiencies in care.^4^ In the US, pregnant people with SCD face intersectional risks, as most are members both of an understudied genetic disease community and an historically marginalized racial group. In studies involving the nonpregnant population, SCD and racial disparities are also known to act synergistically in driving adverse outcomes.^4,5,6^

Since the last nationally representative report of SCD pregnancy outcomes, which used data from 1999 to 2008, SCD and high-risk pregnancy care has evolved. A growing number of people with SCD survive into their reproductive years due to improvements in supportive care and disease-modifying therapy.^7^ As a consequence of these changes, some affected individuals may be entering their reproductive years with less cumulative end-organ damage, but improved childhood survival may also enable more people with severe SCD phenotypes to pursue pregnancy. Between 2004 and 2014, stronger guidelines addressing prevention of pregnancy-associated thrombosis and hypertensive disorders of pregnancy were released^8,9^; as thrombotic events and hypertensive disorders of pregnancy are common in SCD pregnancy, these advances may have benefited women with SCD. However, whether rates of morbidity and mortality in pregnant people with SCD have improved is unknown.

Contemporary tools to systematically measure pregnancy outcomes are not yet applied to SCD. The US Centers for Disease Control and Prevention (CDC)’s severe maternal morbidity (SMM) index, published in 2012, is a rigorous way to capture highly morbid pregnancy complications in administrative data sets.^10^ The SMM index allows comparison of outcome rates over time and across populations.^11^ The SMM index has not yet been applied to the SCD pregnancy population and may serve as a mechanism to standardize outcomes. People with SCD in the US are approximately 90% Black.^4^ In the US, racial disparities are recognized risk factors for SMM and other adverse outcomes, mediated through socioeconomic inequities, chronic stress, and interpersonal racism.^11,12,13^ As national efforts to address racial disparities in pregnancy outcomes progress, defining the extent to which these efforts might benefit pregnant people with SCD is necessary.

This study’s primary objective was to use the CDC’s SMM index to tabulate outcomes in SCD deliveries and to compare outcomes with deliveries among Black people and a control sample without SCD or Black race using the current US-based National Inpatient Sample (NIS). The secondary objective was to measure racial disparities as a modifier of SMM in pregnancies among people with SMM. We hypothesized that these pregnancies would remain high-risk compared with the pregnancies of people without SCD, and that SMM risk in SCD would be partially attributable to racial disparities.^1,2,14^

## Methods

### Data Set

This cross-sectional study was approved by the Johns Hopkins institutional review board and adhered to the Strengthening the Reporting of Observational Studies in Epidemiology (STROBE) reporting guidelines. Informed consent was not required because data were deidentified. We used data from the 2012-2018 NIS, which includes data from all years since the NIS’s 2012 sampling redesign. The NIS is a 20% stratified sample of all discharges from US hospitals, excluding long-term care facilitates and federally managed hospitals, and is managed by the Agency for Healthcare Research and Quality.^14^ As of 2012, the sample reflected 97% of the US population. NIS admissions are fully deidentified and consequently patients cannot be linked across admissions. Data were analyzed from September 2021 to August 2022.

### Inclusion Criteria

We identified pregnancy deliveries by *International Classification of Diseases, Ninth Revision* (*ICD-9*) and *International Statistical Classification of Diseases and Related Health Problems, Tenth Revision* (*ICD-10*) codes as previously described,^15^ and included deliveries to people aged 11 to 55 years (eTable 1 in Supplement 1). We excluded admissions for which diagnosis data was missing. We also excluded admissions containing codes for miscarriage, which are often managed in the outpatient setting and cannot be reliably studied using an inpatient database. After describing the sample demographics, we excluded deliveries with codes for multifetal gestation, which confound interpretation of SMM.

### Outcomes

We measured SMM using the CDC’s SMM index, which includes 21 highly morbid obstetric complications. Transfusion and sickle cell vaso-occlusive crisis are 2 of the 21 complications in the SMM index, and we excluded them from the analysis of SMM due to confounding by SCD. The final analytic models therefore included 19 of 21 SMM outcomes, considered individually and as a composite SMM variable (eTable 1 in Supplement 1). We analyzed additional adverse outcomes: gestational hypertension, preeclampsia, placental abruption, preterm premature rupture of membranes, cesarean delivery, instrumented vaginal delivery, preterm delivery, peripartum infection (urinary tract infection, endomyometritis, wound infection, or sepsis), postpartum hemorrhage, intrauterine growth restriction, and intrauterine fetal demise. Although we excluded transfusion in the SMM analysis, transfusion was analyzed as an independent secondary outcome.

### Delivery Analysis Groups

We defined 3 analytic groups that were compared in this analysis. The first was SCD deliveries, which included those with at least 1 SCD diagnosis code and without any sickle cell trait code. This approach had a greater than 90% positive predictive value for identifying individuals with SCD.^16^ The second, Black race deliveries, included delivery admissions without an SCD diagnosis code and to pregnant people identified as Black by the NIS’s race variable. Race is not biological and is a proxy for exposure to historical and contemporary inequalities with measurable effects on health outcomes.^12^ In the NIS, the race variable includes both race and ethnicity identifiers, which are drawn from discharge records from source hospitals. Thus, the method by which maternal racial classification was determined for each pregnancy varies based on hospital protocols. The third group in our analysis, non-Black control deliveries, included delivery admissions among people with no SCD diagnosis code and identified as any race except Black by the NIS’s race variable.

### Statistical Analysis

#### Primary Analyses

We compared descriptive characteristics and rates of SMM and other adverse outcomes in deliveries to people with SCD with those among people with Black race and the non-Black control group using *t* tests or χ^2^ tests. We compared SMM in SCD and Black race deliveries to non-Black control deliveries using multiple logistic regression models that estimated adjusted odds ratios (aOR) and 95% CIs.

Patient and hospital characteristics were included as variables in multiple logistic regression models if they were significantly different between analysis groups, associated with SMM, and fit plausibly into a potential causal pathway as confounders or covariates. Final multiple logistic regression models adjusted for patient’s age, public insurance status, and income quartile by zip code, as well as for hospital volume, teaching status, ownership, and regional location. Models did not adjust for race because race was intrinsic to the models. Because race is a covariate in the association between SCD and SMM and other outcomes, we conducted a sensitivity analysis in which we used multiple logistic regression to compare Black race deliveries with and without SCD (eTable 4 in Supplement 1).

#### Secondary Analyses

In secondary analyses, we estimated the proportion of the risk for SMM and other outcomes in SCD deliveries attributable to the group’s majority Black racial composition. This analysis was performed using a population-level analysis called an excess risk analysis.^17^ Excess risk analysis is a method that compares the scenario described by the data set with a counterfactual scenario in which the SCD deliveries were entirely among non-Black people.^4^ Outcomes were estimated by logistic regression for each scenario using data from the NIS data set. Estimates for the 2 scenarios were compared. The difference in risk was the extent to which racial disparities explained SMM and other adverse outcomes in SCD pregnancies in this sample.

All analyses were performed with Stata SE version 17.0 (StataCorp LLC). Missing data were handled with listwise deletion for each model, in accordance with standards issued by the data set administrators and implemented in previous studies using the NIS (eTable 2 in Supplement 1).^18^ All statistical tests were survey-weighted. To minimize the risk of false discovery, we applied the Benjamini and Yekutieli correction^19^ across all analyses, and used a tolerable false discovery rate of less than 5%. All reported *P* values were corrected, and comparisons reported as significant had *P* < .05 in 2-sided tests after multiple comparisons correction.

## Results

### Demographic and Hospital Characteristics for Pregnancy-Related Admissions

The sample included a total 5 401 899 deliveries. There were 3901 deliveries among people with SCD and 742 164 deliveries among birthing people with Black race (Table 1). As expected, the majority of SCD deliveries (3200 [84.0%]) were to Black pregnant people. Compared with the non-Black control deliveries group, deliveries to people in the SCD and Black race samples had similar characteristics, including younger age at delivery (mean [SD] age: SCD, 27.3 [5.9]; Black race, 27.1 [6.1] years; *P* = .38; control, 28.7 [5.9] years; *P* < .001), higher public insurance rates (SCD, 2609 [67.3%]; Black race, 496 828 [65.4%]; *P* = .10; non-Black control, 1 880 198 [40.8%]; *P* < .001), and a higher proportion of households in zip codes below the median income (SCD, 2686 [70.0%]; Black race, 545 558 [72.7%]; *P* < .001; non-Black control, [50.5%]; *P* < .001). Compared with deliveries to the non-Black control and Black race groups, deliveries to people with SCD were more likely to occur at urban teaching hospitals (SCD, 3149 [80.5%]; Black race, 555 026 [72.8%]; *P* < .001; non-Black control, 2 813 699 [60.5%]; *P* < .001), had the longest delivery admission length of stay (mean [SD] stay: SCD, 4.4 [5.0] days; Black race, 2.9 [2.9] days; *P* < .001; non-Black control, 2.6 [2.3] days; *P* < .001), and had the highest rate of multiple gestation (SCD, 154 deliveries [4.0%]; Black race, 19 091 deliveries [2.5%]; *P* < .001; non-Black control, 93 134 [2.0%]; *P* < .001).

**Table 1.** Descriptive Characteristics of Delivery Admissions Among SCD, Black Race, and Non-Black Control Delivery Groups

Characteristics	Deliveries, No. (%)		*P* value, Black race vs SCD^a^	Non-Black control deliveries, No. (%) (n = 4 545 015)	*P* value, Non-Black control vs SCD^a^
	SCD (n = 3747)	Black race (n = 742 164)			
Age, mean (SD), y	27.2 (5.9)	27.1 (6.1)	.27	28.7 (5.9)	<.001
Patients aged ≥35 y	522 (13.5)	101 072 (13.2)	.85	792 500 (16.9)	<.001
Race					
Black	3200 (84.0)	761 255 (100)	<.001	0	<.001
Hispanic	246 (6.5)	NA		1 059 451 (24.6)	
White	158 (4.2)	NA		2 692 348 (62.0)	
Other^b^	200 (5.3)	NA		579 590 (13.4)	
Household income by zip code, $					
1-24 999	1775 (46.2)	372 963 (49.7)	<.001	1 137 855 (24.9)	<.001
25 000-34 999	911 (23.8)	172, 595 (23.0)		1 171 212 (25.6)	
35 000-44 999	698 (18.2)	128 430 (17.1)		1 183 059 (25.8)	
≥45 000	447 (11.8)	77 114 (10.2)		1 085 856 (23.6)	
Public insurance	2609 (67.3)	496 828 (65.4)	.05	1 880 198 (40.8)	<.001
Teaching status					
Urban			<.001		<.001
Teaching	3149 (80.5)	555 026 (72.8)		2 813 699 (60.5)	
Nonteaching	647 (16.8)	161 923 (21.4)		1 341 823 (29.0)	
Rural	105 (2.8)	44 306 (5.8)		482 627 (10.5)	
Hospital volume					
Small	419 (11.0)	103 080 (13.6)	<.001	768 195 (16.7)	<.001
Medium	1076 (27.7)	242 671 (32.0)		1 389 591 (30.0)	
Large	2406 (61.4)	415 504 (54.4)		2 480 363 (53.3)	
Multiple gestation	154 (4.0)	19 091 (2.5)	<.001	93 134 (2.0)	<.001
Length of stay, mean (SD), d	4.4 (5.0)	2.9 (2.9)	<.001	2.6 (2.3)	<.001

Abbreviation: SCD, sickle cell disease.

^a^ Listed *P* values are corrected and derived from comparisons with SCD deliveries.

^b^ Included Asian or Pacific Islander, Native American, and other as defined by each contributing hospital.

Maternal mortality was highest for deliveries among people with SCD: the death rate per 10 000 deliveries was 13.3 (95% CI, 5.7-31.2) for SCD deliveries vs 1.2 (95% CI, 1.0-1.5) among people with Black race and 0.5 (95% CI, 0.5-0.6) among non-Black control deliveries. We considered adverse outcomes in 3 categories: hypertensive disorders of pregnancy, labor and delivery complications, and hematologic and immunologic complications (Table 2). Among hypertensive disorders of pregnancy, the SCD delivery group had the highest rate of each outcome, a gap that increased with increasing outcome severity. Compared with non-Black control deliveries, the odds of SCD deliveries were 1.76 (95% CI, 1.63-1.89) for any hypertensive disorder of pregnancy, 2.43 (95% CI, 2.22-2.66) for preeclampsia, and 4.91 (95% CI, 2.79-8.65) for eclampsia. Among labor and delivery complications, SCD deliveries had higher rates of cesarean delivery (risk ratio [RR]: SCD vs Black, 1.13; 95% CI, 1.08-1.18; SCD vs control, 1.24; 95% CI, 1.19-1.29) and postpartum hemorrhage (SCD vs Black, 1.66; 95% CI, 1.46-1.88; SCD vs control, 1.66; 95% CI, 1.46-1.89) than Black or non-Black control deliveries, but rates of other adverse outcomes in this category were lower in SCD deliveries or did not differ across groups. Among the hematologic and immunologic complications, people with SCD had much higher rates of each adverse outcome.

**Table 2.** Rates of Prevalent Adverse Pregnancy Outcomes in Each Analysis Group

Characteristics	Deliveries, No. (%)		Risk ratio, SCD vs Black	Non-Black control deliveries, No. (%)	Risk ratio, SCD vs control
	SCD	Black			
Any hypertensive disorders of pregnancy	571 (15.24)	90 698 (12.22)	1.25 (1.16-1.34)	394 325 (8.7)	1.76 (1.63-1.89)
Preeclampsia	408 (10.9)	53 248 (7.17)	1.52 (1.39-1.66)	203 807 (4.5)	2.43 (2.22-2.66)
Eclampsia	12 (0.3)	993 (0.13)	2.39 (1.36-4.23)	2966 (0.1)	4.91 (2.79-8.65)
Cesarean delivery	1421 (37.9)	249 285 (33.6)	1.13 (1.08-1.18)	1 389 645 (30.6)	1.24 (1.19-1.29)
Postpartum hemorrhage	217 (5.8)	25 965 (3.5)	1.66 (1.46-1.88)	158 395 (3.5)	1.66 (1.46-1.89)
Instrumented delivery	124 (3.3)	223 477 (4.2)	0.78 (0.66-0.93)	223 477 (4.9)	0.67 (0.57-0.80)
Preterm premature rupture of membranes	113 (3.0)	27 756 (3.7)	0.81 (0.67-0.97)	143 322 (3.2)	0.96 (0.80-1.15)
Placental abruption	50 (1.3)	10 596 (1.4)	0.93 (0.71-1.24)	45 989 (1.0)	1.32 (1.00-1.74)
Transfusion	693 (18.5)	13 213 (1.8)	10.39 (9.67-11.16)	46 707 (1.0)	18.00 (16.73-19.36)
Any peripartum infection	438 (11.7)	38 913 (5.2)	2.23 (2.04-2.44)	173 342 (3.8)	3.07 (2.80-3.36)
Venous thromboembolism	36 (1.0)	526 (0.1)	13.56 (9.51-19.32)	2201 (0.1)	19.84 (14.00-28.12)

Abbreviation: SCD, sickle cell disease.

### High SMM and Adverse Fetal Outcome Rates in SCD Deliveries

We measured the prevalence of SMM in each group (eTable 3 in Supplement 1). Compared with the other groups, the SCD delivery sample had higher per admission composite SMM rates (SCD, 5.7% vs Black, 1.1%; *P* < .001; vs 0.7% non-Black control; *P* < .001).

**Figure 1.**
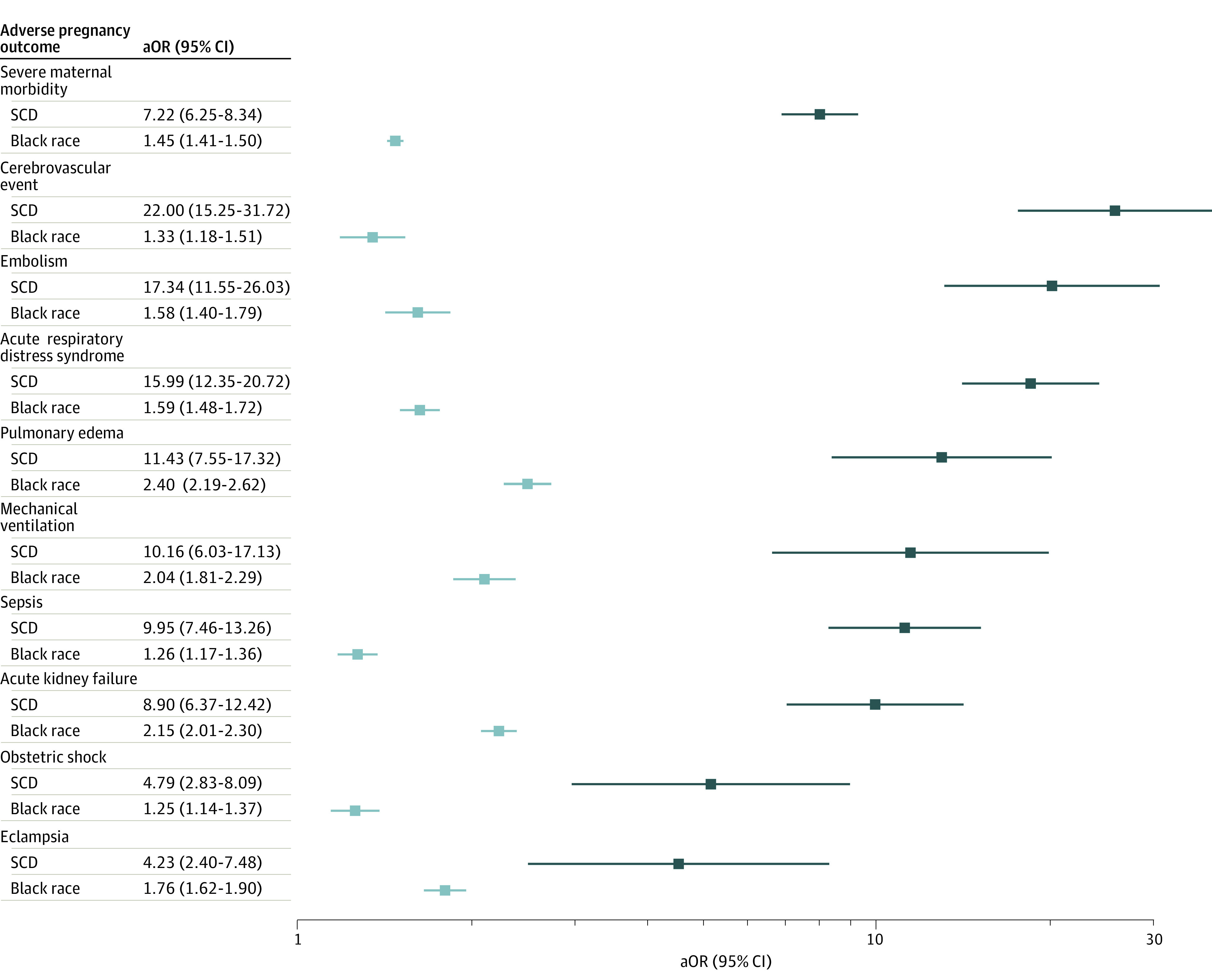
Adjusted Odds of Severe Maternal Morbidity (SMM) Among Sickle Cell Disease (SCD) and Black Race Deliveries Compared With Non-Black Control Deliveries All adjusted odds ratios (aORs) were significant after using the Benjamini-Yekutieli correction. aORs adjusted for patient age, public insurance status, and income quartile by zip code, as well as hospital geographical region, bed size, teaching status, and ownership.

Adjusted odds of SMM complications were calculated using multivariable models that included patient’s age, public insurance status, and income quartile by zip code, as well as hospital geographical region, bed size, teaching status, and ownership (Figure 1). Compared with deliveries to the non-Black control group, the adjusted odds of SMM were higher among people with SCD than deliveries to Black people (adjusted odds ratio [aOR]: SCD, 7.22; 95% CI, 6.25-8.34; Black race, 1.45; 95% CI, 1.41-1.50). SCD deliveries had increased odds of each SMM index measure above both Black race and non-Black control deliveries, though the OR was insignificant for the rarest events (heart failure and myocardial infarction) and SCD deliveries had no instances of amniotic fluid embolism or aneurysm. Compared with non-Black control deliveries, SCD deliveries had especially elevated odds of cerebrovascular events (aOR, 22.00; 95% CI, 15.25-31.72) and air or thrombotic embolism (aOR, 17.34; 95% CI, 11.55-26.03).

**Figure 2.**
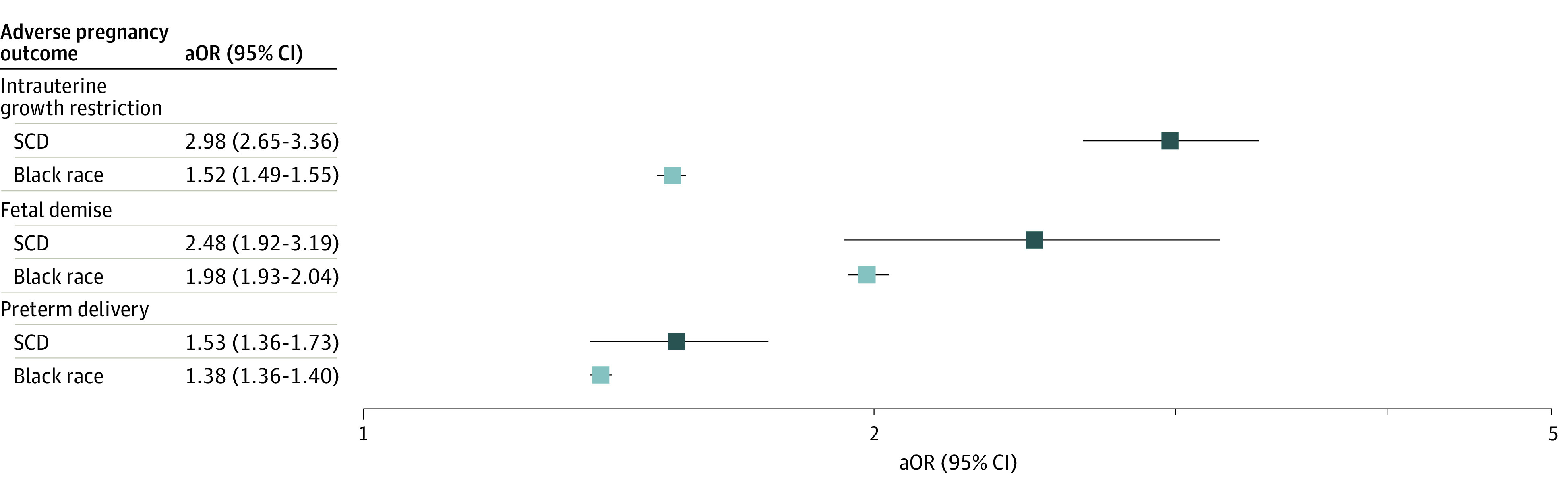
Adjusted Odds of Adverse Fetal Outcomes Among Sickle Cell Disease (SCD) and Black Race Deliveries Compared With Non-Black Control Deliveries All adjusted odds ratios (aORs) were significant after using the Benjamini-Yekutieli correction. aORs adjusted for patient age, public insurance status, and income quartile by zip code, as well as hospital geographical region, bed size, and teaching status.

Compared with the non-Black control deliveries group, deliveries to pregnant people with SCD had increased risk of intrauterine growth restriction, intrauterine fetal demise, and preterm delivery (Figure 2). The SCD deliveries group had higher odds of intrauterine growth restriction than Black race deliveries (SCD: aOR, 2.98; 95% CI, 2.65-3.36; Black race: aOR, 1.52; 95% CI, 1.49-1.55). However, SCD and Black race deliveries had no difference in the associated odds of fetal demise (SCD: aOR, 2.48; 95% CI, 1.92-3.19; Black race: aOR, 1.98; 95% CI, 1.93-2.04) or preterm delivery (SCD: aOR, 1.53; 95% CI, 1.36-1.73; Black race: aOR, 1.38; 95% CI, 1.36-1.40).

### Excess Risk of SCD Pregnancy Complications Attributable to Racial Disparities

To assess how racial disparities contributed to risk of SMM and other adverse outcomes, we performed an excess risk analysis focusing on pregnancy complications among SCD deliveries attributable to racial disparities (Table 3). The median excess risk (ER) attributable to racial inequities was 28.9% (IQR, 21.2%-33.1%). Racial disparities accounted for half of the increased risk in SCD deliveries for acute kidney failure (ER, 56.9%; 95% CI, 54.3%-59.3%), intrauterine fetal demise (ER, 47.8%; 95% CI, 46.6%-49.1%) and eclampsia (ER, 42.1%; 95% CI, 37.9%-46.1%). Deliveries to Black people and people with SCD had elevated risk of cesarean delivery compared with the non-Black control sample, and only a small proportion of the increased risk for cesarean delivery risk among SCD deliveries was attributable to racial disparities (ER, 8.8%; 95% CI, 8.3%-9.2%).

**Table 3.** Proportion of Adverse Outcomes Among SCD Deliveries Attributable to Racial Disparities

Characteristic	Risk difference between SCD and non-Black control deliveries, % (95% CI)	Risk ratio between SCD and non-Black control deliveries (95% CI)	ER, % (95% CI)^a^
Severe maternal morbidity	5.0 (4.2 to 5.7)	7.9 (6.9 to 9.1)	33.1 (31.5 to 34.7)
Hypertensive disorder of pregnancy			
Eclampsia	0.3 (0.1 to 0.4)	4.9 (2.8 to 8.6)	42.1 (37.9 to 46.1)
Preeclampsia	6.4 (5.4 to 7.4)	2.4 (2.2 to 2.7)	30.0 (29.1 to 30.9)
Composite hypertensive disorders	6.6 (5.4 to 7.7)	1.8 (1.6 to 1.9)	21.2 (20.4 to 22.2)
Labor and delivery associated complications			
Preterm delivery	3.3 (2.4 to 4.1)	1.7 (1.6 to 1.9)	27.1 (26.2 to 28.0)
Placental abruption	0.3 (−0.1 to 0.7)	1.3 (1.0 to 1.7)	26.0 (24.4 to 27.6)
Cesarean delivery	7.3 (5.7 to 9.0)	1.2 (1.2 to 1.3)	8.8 (8.3 to 9.2)
Postpartum hemorrhage	2.3 (1.6 to 3.0)	1.7 (1.5 to 1.9)	4.7 (3.2 to 6.3)
Complications throughout pregnancy and the postpartum period			
Acute kidney failure	1.0 (0.6 to 1.3)	12.0 (8.7 to 16.6)	56.9 (54.3 to 59.3)
Cerebrovascular event	0.9 (0.5 to 1.2)	26.7 (18.4 to 37.8)	28.9 (21.1 to 35.8)
Venous thromboembolism	0.9 (0.6 to 1.3)	20.0 (14.0 to 28.1)	29.4 (22.9 to 35.4)
Composite infection	7.9 (6.8 to 8.9)	3.1 (2.8 to 3.4)	19.0 (17.7 to 20.3)
Fetal complication			
Intrauterine fetal demise	1.0 (0.6 to 1.4)	2.7 (2.1 to 3.4)	47.8 (46.6 to 49.1)
Intrauterine growth restriction	5.9 (5.0 to 6.8)	3.3 (2.9 to 3.6)	28.8 (27.6 to 29.9)

Abbreviations: ER, excess risk; SCD, sickle cell disease.

^a^ ER describes the proportion of risk difference in SCD deliveries explained by racial disparities.

## Discussion

Individuals with SCD face markedly higher risk of SMM and other adverse outcomes than the general pregnant population. Deliveries to people with SCD were 5 times more likely to have SMM compared with Black pregnant people, and 7 times more likely to have SMM than the non-Black control group. Maternal mortality in the SCD delivery group was 10 times greater than in the Black delivery group and 26 times greater than in the non-Black control. In this study, the rates of adverse pregnancy outcomes were equal to or greater than the rates reported in 15-to-25-year-old data.^1,2^ In data from 2000 to 2003, the maternal mortality rate among people with SCD was 7.2 deaths per 10 000.^1^ In this study, we report rates of 13.3 deaths per 10 000 deliveries. Maternal mortality in SCD is not improving. These findings suggest that advancements in SCD and high-risk obstetric care are not reaching pregnant people with SCD.

While evidence has emerged to inform the management of high-risk pregnancy in general, few rigorously studied SCD-specific management recommendations exist, despite longstanding recognition of unique risks. At present, when and how to use SCD treatments during pregnancy is not established. By 2022, the American College of Obstetricians and Gynecologists recommended low-dose aspirin use in women with SCD starting at 12-weeks gestation,^20^ and a clinical trial of low-dose aspirin to prevent preeclampsia in SCD is ongoing.^21^ There is not yet disease-specific evidence to guide peripartum or postpartum anticoagulation use in pregnant people with SCD without a history of thrombosis. A clinical trial is assessing the feasibility of chronic transfusion in SCD pregnancy (NCT03975894).^22^ Rigorous, standardized studies of clinical and structural pathways to improve outcomes are needed.^23^

The elevated risk of SMM and other adverse outcomes in SCD is partly attributable to racial disparities in maternal morbidity and mortality.^24^ Here, increased rates of SMM and other adverse outcomes in SCD were partially attributable to racial disparities. Our analysis suggests that approximately 50% of the increased risk of acute kidney failure, intrauterine fetal demise, and eclampsia among deliveries to people with SCD could be eliminated by addressing racial disparities in maternal health. Recognition of racial disparities in pregnancy outcomes compels efforts to improve access to comprehensive medical care, transportation, housing, and support services, which could meaningfully improve outcomes in SCD pregnancies.^23^

How complex SCD pathophysiology contributes to adverse pregnancy outcomes is understudied. The chronic hemolytic anemia of SCD causes vasculopathy and angiogenesis, which may contribute to placental abnormalities that are associated with intrauterine growth restriction, hypertensive disorders of pregnancy, and miscarriage, all of which are common in SCD pregnancies.^25,26^ SCD pregnancies are almost universally exposed to maternal anemia, which drives adverse maternal and fetal outcomes in the non-SCD population. The effects of maternal anemia on immediate or long-term SCD pregnancy outcomes are not established.^27,28^ SCD and pregnancy are hypercoagulable states. How the hyperestrogenism of pregnancy affects the baseline hypercoagulability in SCD is not established.^29^

The failure to improve outcomes in pregnant people with SCD, despite long recognized risk, reflects profound deficiencies in SCD clinical and research structures.^5,24,30^ In high- and low-income settings, more people with SCD are surviving into their reproductive years.^31^ Although SCD pregnancy has been recognized as high-risk for over 50 years, disease-specific studies of SCD pregnancy pathophysiology and of interventions to affect pregnancy outcomes are limited or inconclusive.^25,32,33^ This study relies on billing codes because no contemporary registry yet contains the obstetric data needed to analyze a nationally representative sample of pregnant people with SCD. Yet, the evidence that such a study might be transformative is clear, given the field-defining output from the Cooperative Study of Sickle Cell Disease (conducted 1977 to 1998).^34^ Additionally, studies addressing the structural and biological causes of high maternal morbidity and mortality in SCD are needed. SCD research receives lower levels of federal and foundation funding compared with less common diseases,^35^ and SCD pregnancy research needs organizational, institutional, pharmaceutical, and federal support.

This study used the redesigned NIS, which represents a more uniform sample of US hospital admissions than the previously available design.^14^ A 2016 SCD pregnancy study in the US limited their focus to single-center or single-state analyses, while ours was nationally representative.^36^ We quantified outcomes using the CDC’s SMM index, which supports integration of diverse data sets and which may help benchmark secular trends in outcomes over time as research progresses. We also considered racial disparities as an adverse exposure in SCD pregnancy.

### Limitations

This study had several limitations. First, analysis was limited by reliance on an administrative data set. The NIS does not provide reliable information about parity, obesity, or smoking, so these risks could not be analyzed. *ICD* codes unreliably identify SCD genotype,^37^ and genotype-specific outcomes are needed to individualize care. NIS admissions are not linked to individuals, so pregnancies cannot be followed across admissions to observe temporal relationships between exposures and outcomes. The NIS does not include information related to outpatient prenatal care or the involvement of subspecialty consultants in a patient’s care; expert care is shown to affect certain outcomes in SCD pregnancy.^38^ The NIS data set does not capture out-of-hospital births, which may include less complicated pregnancies, and excludes admissions to federal hospitals like the Veterans Affairs system, although together these groups account for about 1.5% of deliveries in the US. In the NIS, information about race is not missing at random so we were unable to impute missing data and relied on listwise deletion.^39,40^ However, the rate of missing data for race was only 5.7% for all admissions, and the rate of missing data was even lower for all other variables. Ultimately, the NIS draws from the majority of births in the US, providing unmatched sample size and generalizability for epidemiologic data.

## Conclusions

In this representative cross-sectional sample of pregnancies in the US, pregnancy in people with SCD were at very high risk, with mortality rates among SCD deliveries 26 times the general population. Despite advances in SCD management and high-risk pregnancy care, at the national level, outcomes in this population have not improved since the last NIS analysis of data from 1999 to 2008.^1,2^ However, disease-specific interventions will also be required to mitigate the enduring SMM we identify. This too must be understood as a racial health care disparity, as failures to advance SCD research and care in the US are also consequences of structural racism.^24,39^ While our results show failure to improve outcomes for this population, they also provide a baseline using a replicable measurement tool, the CDC’s SMM index, against which to compare outcomes in the future. Our findings compel focused scientific, clinical, and political effort to improve outcomes for pregnant people with SCD.

## Notes

Supplement 1. **eTable 1.** *ICD-9* and *ICD-10* Codes Used to Identify Independent and Dependent Variables **eTable 2.** Proportion of Missing Data, by Variable **eTable 3.** Survey-weighted Rates of Composite Severe Maternal Morbidity and Component Events in Delivery Admissions, by Group **eTable 4.** Comparative Results of Sensitivity Analysis: Minimal Meaningful Change in Risk When Considering Only Admissions Coded With Black Race

Supplement 2.
Data Sharing Statement
