# Supplementary material for: Severe Maternal Morbidity and Mortality in Sickle Cell Disease in the National Inpatient Sample, 2012-2018
Source: JAMA Netw Open. 2023 Feb 2;6(2):e2254552. doi: 10.1001/jamanetworkopen.2022.54552 (PMC9896307; doi:10.1001/jamanetworkopen.2022.54552)
Supplement: Supplement 1. — eTable 1. ICD-9 and ICD-10 Codes Used to Identify Independent and Dependent Variables eTable 2. Proportion of Missing Data, by Variable eTable 3. Survey-weighted Rates of Composite Severe Maternal Morbidity and Component Events in Delivery Admissions, by Group eTable 4. Comparative Results of Sensitivity Analysis: Minimal Meaningful Change in Risk When Considering Only Admissions Coded With Black Race [file jamanetwopen-e2254552-s001.pdf]

# Supplemental Online Content

Early ML, Eke AC, Gemmill A, Lanzkron S, Pecker LH. Severe maternal morbidity and mortality in sickle cell disease in the national inpatient sample, 2012-2018. *JAMA Netw Open.* 2023;6(2):e2254552. doi:10.1001/jamanetworkopen.2022.54552

**eTable 1.** *ICD-9* and *ICD-10* Codes Used to Identify Independent and Dependent Variables

**eTable 2.** Proportion of Missing Data, by Variable

**eTable 3.** Survey-weighted Rates of Composite Severe Maternal Morbidity and Component Events in Delivery Admissions, by Group

**eTable 4.** Comparative Results of Sensitivity Analysis: Minimal Meaningful Change in Risk When Considering Only Admissions Coded With Black Race

This supplemental material has been provided by the authors to give readers additional information about their work.

© 2023 Early ML et al. *JAMA Network Open.*

**Supplemental Table 1:** ICD-9 and ICD-10 codes used to identify independent and dependent variables

| Variable | ICD-9 | ICD-10 |
|---|---|---|
| **Descriptive variables** | | |
| Delivery admissions | DX: V27, 650 – 669<br>PR: 72-75 | DX: O60 – O82, Z37-Z38<br>PR: 10D07Z3 – 10D07Z8, 10D00Z0 – 10D00Z2, 10E0XZZ, 0DQR0ZZ, 0HQ9XZZ, 0KQM0ZZ, 0W8NXZZ |
| **Exposures** | | |
| Sickle cell disease | 282.60 – 282.69 or 282.41 – 282.42 | D57.0 – D57.2*, D57.4*, D57.8* |
| **Pregnancy outcomes** | | |
| Acute heart failure[a] | 997.1 | I97.12*, I97.13*, I19.71* |
| Acute myocardial infarction | 410.* | I21.01, I21.02, I21.09, I21.11, I21.19, I21.21, I21.29, I21.3, I21.4, I21.9 |
| Acute renal failure[a] | 584.5, 584.6, 584.7, 584.8, 584.9, 669.3* | N17.0, N17.1, N17.2, N17.8, N17.9, O90.4 |
| Adult respiratory distress syndrome[a] | 518.5*, 518.81, 518.82, 518.84, 799.1 | J80, J95.1, J95.2, J95.3, J95.821, J95.922, J96.00, J96.01, J96.02, J96.20, J96.21, J96.22, R09.2 |
| Air or thrombotic embolism[a] | 415.1*, 673.0*, 673.2*, 673.3*, 673.8* | I26.*, O88.0*, O88.2*, O88.3*, O88.8* |
| Amniotic fluid embolism[a] | 673.1* | O88.11*, O88.12, O88.13 |
| Aneurysm[a] | 441.* | I71.00-I71.03, I71.1, I71.2, I71.3, I71.4, I71.5, I71.6, I71.8, I71.9, I79.0 |
| Cardiac arrest[a] | 427.41, 427.42, 427.5 | I46.2, I46.8, I46.9, I49.0* |
| Cerebrovascular event[a] | 430.0 – 437.99, 671.5*, 674.0*, 997.02 | I60 – I68.8, O22.51, O22.52 I97.81*, I97.82*, O87.3 |
| Cesarean delivery | DX: 669.7<br>PR: 74.0, 74.1, 74.2, 74.4, 74.9* | DX: O82*, O75.82, z3801, z3831, z3862, z3864, z3866, z3869<br>PR: 10D00Z0-10D00Z1 |
| Conversion of cardiac rhythm[a] | 99.6* | 5A2204Z, 5A12012 |
| Disseminated intravascular coagulation[a] | 286.6, 286.9, 666.3* | D65, D68.8, D68.9, O72.3 |
| Eclampsia[a] | 642.6* | O15.0*, O15.1, O15.2, O15.9 |
| Hypertensive disorder of pregnancy, composite | 642.3* 642.4* 642.5* 642.6* 642.7* | O11* O13* O14* O15* |
| Hysterectomy[a] | PR: 68.3* - 68.9* | PR: 0UT90ZZ, 0UT94ZZ, 0UT97ZZ, 0UT98ZZ, 0UT9FZZ |

© 2023 Early ML et al. *JAMA Network Open.*

| | | |
|---|---|---|
| Instrumented vaginal delivery | DX: 669.5<br>PR: 72* | PR: 10D07Z3, 10D07Z4, 10D07Z5, 10D07Z6, 10D07Z7 |
| Intrauterine fetal demise | 656.4*, V27.1, V27.3, V27.4, V27.6, V27.7 | O36.4*, Z37.1, Z37.3, Z37.4, Z37.6, Z37.7 |
| Intrauterine growth restriction | 656.5* | O36.5* |
| Peripartum infection | 646.5*, 646.6*, 658.4, 659.3*, 670*, 672*, 674.1*, 674.2*, 674.3* | O23.0*, O23.1*, O23.2*, O23.3*, O23.4*, O41.1*, O75.3*, O85*, O86.0*, O86.1*, O86.2*, O86.4*, O86.8*, O90.0, O90.1 |
| Placental abruption | 641.2* | O45* |
| Post-partum hemorrhage | 666* | O67.0, O67.8, O67.9, O72.0, O72.1, O72.2, O72.3 |
| Preterm delivery | 644.2 | O60.1* |
| Preeclampsia | 642.4*, 642.5*, 642.7* | O11*, O14* |
| Preterm premature rupture of membranes | 658.1 | O42.01*, O42.11*, O42.91* |
| Pulmonary edema[a] | 518.4, 428.1, 428.0, 428.21, 428.23, 428.31, 428.33, 428.41, 428.43 | J81.0, I50.1, I50.20, I50.21, I50.23, I50.30, I50.31, I50.33, I50.40, I50.41, I50.43, I50.9 |
| Sepsis[a] | O38.*, 995.91, 995.92, 670.2* | O85, O86.04, T80.211, T81.4* |
| Severe anesthesia complications[a] | 668.0*, 668.1*, 668.2* | O74.0, O74.1, O74.2, O74.3, O89.01, O89.09, O89.1, O89.2 |
| Shock[a] | 669.1*, 785.5*, 99.0, 995.4, 998.0* | O75.1, R57.*, R65.21, T78.2*, T88.2*, T88.6*, T81.1* |
| Temporary tracheostomy[a] | PR: 31.1 | PR: 0B110Z4, 0B110F4, 0B113Z4, 0B113F4, 0B114Z4, 0B114F4 |
| Transfusion | DX: V58.2<br>PR: 99.0* | PR: 30230H* 30230N* 30230P* 30233H* 30233N* 30233P* 30240H* 30240N* 30240P* 30243H* 30243N* 30243P* |
| Venous thromboembolism | 415.1*, 453.4*, 453.82, 453.83, 453.84, 453.85, 453.86, 453.87, 453.89, 671.3, 671.4, 673.2 | I26*, O22.3*, O87.1, O88.2* |
| Ventilation[a] | PR: 93.90, 96.01, 96.02, 96.03, 96.05 | PR: 5A1935Z, 5A1945Z, 5A1955Z |

[a]Included in the Severe Maternal Morbidity Index; *Includes all codes that have additional digits, as long as digits preceding the asterisk match

© 2023 Early ML et al. *JAMA Network Open.*

**Supplemental Table 2:** Proportion of missing data, by variable

| Variable | Missing SCD % | Missing Black % | Missing control % | Missing total % |
|---|---|---|---|---|
| Race | 2.4 | 0.0 | 6.6 | 5.7 |
| Income quartile by zip code | 1.8 | 1.3 | 1.3 | 1.3 |
| Public insurance | 0.1 | 0.2 | 0.2 | 0.2 |
| Hospital volume | -- | -- | -- | 0 |
| Hospital location | -- | -- | -- | 0 |
| Hospital teaching status | -- | -- | -- | 0 |
| Hospital ownership | -- | -- | -- | 0 |

© 2023 Early ML et al. *JAMA Network Open.*

**Supplemental Table 3:** Survey-weighted rates of composite severe maternal morbidity and component events in delivery admissions, by group

| Complication | Rate, SCD, % | Rate, Black race, % | Rate, control, % |
|---|---|---|---|
| Severe maternal morbidity | 5.68 | 1.12 | 0.72 |
| Acute myocardial infarction | 0.03 | 0.01 | <0.01 |
| Acute pulmonary edema | 0.69 | 0.13 | 0.05 |
| Acute renal failure | 1.07 | 0.22 | 0.09 |
| Acute respiratory distress syndrome | 1.71 | 0.15 | 0.08 |
| Air or thrombotic embolism | 0.64 | 0.05 | 0.03 |
| Amniotic fluid embolism | -- | <0.01 | <0.01 |
| Anesthesia complications | 0.13 | 0.02 | 0.10 |
| Aneurysm | -- | <0.01 | <0.01 |
| Cardiac arrest | 0.16 | 0.02 | 0.01 |
| Cardioversion | 0.13 | 0.02 | 0.01 |
| Cerebrovascular event | 0.88 | 0.05 | 0.03 |
| Disseminated intravascular coagulation | 0.91 | 0.29 | 0.25 |
| Eclampsia | 0.32 | 0.13 | 0.07 |
| Hysterectomy | 0.35 | 0.13 | 0.12 |
| Mechanical ventilation | 0.43 | 0.07 | 0.03 |
| Obstetric shock | 0.37 | 0.09 | 0.07 |
| Sepsis | 1.28 | 0.15 | 0.10 |
| Temporary tracheostomy | 0.05 | 0.01 | <0.01 |

© 2023 Early ML et al. *JAMA Network Open.*

**Supplemental Table 4**: Comparative results of sensitivity analysis: minimal meaningful change in risk when considering only admissions coded with Black race

| | **SCD deliveries versus controls deliveries (all races)** | **SCD deliveries versus control deliveries, restricted only to deliveries with Black race** |
|---|---|---|
| | **SCD,** aOR (95% CI)<br>n=3,901 | **SCD,** aOR (95% CI)<br>n=3,070 |
| **Severe maternal morbidity** | 7.22 (6.25-8.34) | 5.26 (4.49-6.15) |
| **Cerebrovascular event** | 22.00 (15.25-1.72) | 18.76 (12.84-27.39) |
| **Air or thrombotic embolism** | 17.34 (11.55-26.03) | 12.79 (8.24-19.87) |
| **Acute respiratory distress syndrome** | 15.99 (12.35-20.72) | 11.42 (8.67-1.07) |
| **Acute pulmonary edema** | 11.43 (7.55-17.32) | 5.63 (3.59-8.82) |
| **Mechanical ventilation** | 10.16 (6.03-17.13) | 5.98 (3.49-10.25) |
| **Sepsis** | 9.95 (7.46-13.26) | 8.19 (5.94-11.30) |
| **Acute renal failure** | 8.90 (6.37-12.42) | 4.90 (3.45-6.95) |
| **Obstetric shock** | 4.79 (2.83-8.09) | 3.94 (2.18-7.14) |
| **Eclampsia** | 4.23 (2.40-7.48) | 2.69 (1.48-4.88) |
| **Hypertensive disorders of pregnancy** | 1.77 (1.62-1.93) | 1.28 (1.17-1.41) |
| **Preeclampsia** | 2.30 (2.08-2.55) | 1.58 (1.42-1.77) |
| **Cesarean delivery** | 1.47 (1.37-1.57) | 1.27 (1.18-1.37) |
| **Any peripartum infection** | 2.75 (2.47-3.06) | 2.28 (2.03-2.55) |

aORs are adjusted for patient age, public insurance status, and income quartile by zip code; and hospital geographical region, bed size, and teaching status.

© 2023 Early ML et al. *JAMA Network Open.*
