# Supplementary material for: Severe Maternal Morbidity and Mortality in Sickle Cell Disease in the National Inpatient Sample, 2012-2018
Source: JAMA Netw Open. 2023 Feb 2;6(2):e2254552. doi: 10.1001/jamanetworkopen.2022.54552 (PMC9896307; doi:10.1001/jamanetworkopen.2022.54552)
Supplement: Supplement 2. — Data Sharing Statement [file jamanetwopen-e2254552-s002.pdf]

Data Sharing Statement

Early. Severe Maternal Morbidity and Mortality in Sickle Cell Disease in the National Inpatient Sample, 2012-2018. *JAMA Netw Open*. Published February 02, 2023. doi:10.1001/jamanetworkopen.2022.54552

Data
Data available: No

Additional Information
Explanation for why data not available: The data is managed by the Agency for Healthcare Research and Quality and is only available through registration with this entity and completion of their required training module.
